# Books and Pamphlets Received

**Published:** 1886-01-20

**Authors:** 


### BOOKS AND PAMPHLETS RECEIVED.

*A System of Practical Medicine by American Authors.* Edited by Wm. Pepper, M. D., LL. D., Provost and Professor of the Theory and Practice of Medicine, and of Clinical Medicine, in the University of Pennsylvania ; assisted by Louis Starr, M. D., Clinical Professor of Diseases of Children in the University of Pennsylvania. Volume I—Pathology and General Diseases. Philadelphia : Lea Brothers & Co. 1885.

The above work contains 1,094 large octavo pages, neatly and substantially bound in leather, and of elegant typographical execution. "It was undertaken," says the editor, "in the belief that, by obtaining the co-operation of a considerable number of physicians of acknowledged authority, who should treat subjects selected by themselves, there could be secured an amount of practical information and teaching not otherwise accessible." The work is exclusively American, the selection of authors being confined to the United States and

Canada, it being the design to make a comprehensive presentation of the whole field of Medicine as it is taught and practiced by its best representatives in this country. Able and competent men, thoroughly posted in the particular departments of which they wrote, have been selected for the important tasks severally undertaken by them.

The present volume is the first of a series yet to appear, designed to furnish a concise and thoroughly practical system, embracing a very wide field of study. The volume before us contains, first, a series of able articles on General Pathology and Sanitary Science. The writers under this head are Regenald H. Fetz, M.D., Henry Hartshorne, M.D., LL.D., John S. Billings, A.M., M.D., LL.D. and George E. Waring, jr., M.D., I.C.E. The second department of the volume relates to General Diseases, including the various fevers, together with the eruptive fevers, yellow fever, etc.; also, diphtheria, cholera, plague, leprosy, cerebro spinal meningitis, dengue, rabies, anthrax, pyæmia, etc. It is not possible, in our brief space, to detail the excellencies of this great work. It is worthy the attention of practieal and scientific men who desire to keep posted on the advances of the profession, and should be in the library of every intelligent medical man.

*Human Osteology;* comprising a description of the bones, with delineations of the attachments of the Muscles, the general and microscopic structure of Bone, and its development. By Luther Holden. New York: William Wood & Company.

We doubt whether we ever read a medical work with more interest and profit than we did the above. Every line commends itself to the attention of the histiologist, the anatomist, and the surgeon. The work is well gotten up typographically, and is fully and beautifully illustrated.

*Diseases of the Lungs* (of a specific, not tuberculous nature), Acute Bronchitis, Infectious Pneumonia, Gangrene, Syphilis, Cancer, and Hydatid of the Lungs. By Prof. Germain See, Member of the Academy of Medicine; Member of the Society of Medicine; Physician to the Hotel Dieu, Paris, France. Translated by E. P. Hurd, M. D., Member of the Massachusetts Medical Society; Vice-President of the Essex North Medical Society, etc. With Appendices, by George M. Sternberg, M. D., Surgeon U. S. A., and Prof. Dujardin Beaumetz, Member of the Academy of Medicine; Physician to the Hospital Cochin, Paris, etc. New York: William Wood & Co. 1885.

A work of 398 large octavo pages; illustrated, well written, and replete with interest and practical instruction upon the subjects treated.

*Milk Analyses and Infant Feeding;* a practical treatise on the examinations of Human and Cow's Milk, Cream, Condensed Milk, etc., and Directions as to the Diet of Young Infants. By Arthur V. Meigs, M. D., Physician to the Pennsylvania Hospital, and to the Children's Hospital; Fellow of the College of Physicians of Philadelphia, etc. Philadelphia: P. Blakiston, Son & Co., 1012 Walnut street. 1885.

This little work of 100 pages will be found ably written, and containing much matter of interest to the practitioner.

*Hay Fever, and its Successful Treatment* by superficial organic alterative of the Nasal Mucous Membrane. An Essay, read before the Philadelphia Laryngological Society, April, 1885. By Charles E. Lajous, M.D., Instructor of Rhinology and Laryngology in the Post-graduate and Spring Courses, Jefferson Medical College; President of the Philadelphia Laryngological Society, etc. Illustrated with thirteen wood engravings. Philadelphia: F. A. Davies, attorney and publisher, 1217 Filbert street. 1885.

